# COVID-19 Contact Tracing Experience at a Tertiary Healthcare Center in Saudi Arabia

**DOI:** 10.7759/cureus.41919

**Published:** 2023-07-15

**Authors:** Hamna Abdul Muthalib, Alaa Hamad, Javeed Muhammad, Zainab Ifthikar, Esam Albanyan, Suliman Aljumaah, Salem AlGhamdi

**Affiliations:** 1 Pediatric Infectious Diseases, King Faisal Specialist Hospital and Research Centre, Riyadh, SAU; 2 Medicine, Alfaisal University College of Medicine, Riyadh, SAU; 3 Internal Medicine, University Hospitals of Leicester NHS Trust, Leicester, GBR

**Keywords:** contact tracing, healthcare providers, infection, healthcare, saudi arabia, covid-19

## Abstract

Introduction

As of May 2023, the end of the pandemic, the cumulative number of COVID-19 cases reached more than 841,000 cases. Healthcare workers (HCWs) especially have been at the frontline during this pandemic and are at a higher chance of contracting COVID-19. Approximately half of all high-risk exposures were to healthcare personnel with COVID-19. While several tools for contact tracing were developed for the general public, contact tracing for infectious diseases in the healthcare setting is limited, and global testing of HCWs, or in-hospital digital tracing, is not performed in most facilities. The King Faisal Specialist Hospital and Research Center (KFSH&RC) collaborated with the Infection Control and Health Information Technology Affairs (HITA) to create an automated COVID-19 contact tracing tool specifically for HCWs who worked at the institute. This study aims to describe the contact tracing experience at KFSH&RC.

Methods

A retrospective study was conducted to describe the use of an automated tool that was developed to assist in the contact tracing process and that was to be used by KFSH&RC employees who had been in contact with a COVID-19-positive individual. This tool is utilized for the early identification of possible COVID-19 cases and risk stratification of the exposed individuals. The tool can be accessed through the KFSH&RC website; it also collects information about the COVID-19 exposure rate among the different departments such as administration, capital projects/facilities, and healthcare at a tertiary care hospital in Riyadh, Saudi Arabia

Results

The tool has been utilized 7,353 times by contact cases. Approximately 7% of those tested later developed a COVID-19 infection. When assessing the positivity rates per department, The Environmental Services Department had the highest positivity rate of 28.21%, followed by Health Information Technology and Analytics (HITA), and then the Central Transportation Department.

Conclusion

This study acts as the first of its kind to describe the successful use of the healthcare contact tracing system in one of Saudi Arabia's largest hospitals (KFSH&RC) and describe the infection trends in different departments of the hospital. Through the tracing system, the departments with the highest COVID-19 infection occurrences at the hospital were identified in a timely manner, and safety protocols were implemented.

## Introduction

The first documented COVID-19 case emerged in Wuhan, China in late December 2019, after which it spread to the rest of the world [1]. The first COVID-19 case in Saudi Arabia appeared on March 2nd in the eastern region and spread to the rest of the country shortly afterward [2]. By the end of the pandemic, and as of May 2023, the cumulative number of COVID-19 cases reached more than 841,000 cases [3]. As a result of the overwhelming number of affected cases, healthcare resources, and staff were severely exhausted [4]. Healthcare workers (HCWs) especially have been at the frontline during this pandemic and are at a higher chance of contracting COVID-19. Approximately half of all high-risk exposures were to healthcare personnel with COVID-19 [5,6]. In many countries, including Saudi Arabia, data regarding the infection rate of COVID-19 among HCWs is limited. In Qatar, a study tested 16,912 HCWs for COVID-19, of which 10.6% tested positive; 95% of affected HCWs worked in a non-COVID-19 setting, and 5% acquired the infection at a COVID facility [7]. Among the non-COVID-19 sites, 45% of HCWs reported exposure to a colleague, whereas 29% reported exposure to a patient with COVID-19 [7]. In Saudi Arabia, the prevalence of COVID-19 among HCWs is yet to be studied. Major risk factors for COVID-19 infection among HCWs include inadequate use or unavailability of personal protective equipment (PPE), uncertain diagnostic criteria, or unavailability of diagnostic tests [5].

Contact tracing methods for HCWs in Saudi Arabia

Contact tracing applications have been developed in Saudi Arabia for the general public in response to the COVID-19 pandemic like several other countries around the world; they were successful in tracing cases and preventing them from spreading the infection [8]. However, due to the high-risk nature of HCWs, healthcare facilities also began developing independent contact tracing systems to identify HCWs who have had exposure to a confirmed COVID-19 case and identify at-risk individuals based on the risk classification of low and high. Low-risk HCWs are those who had an exposure while being adequately protected with complete PPE during an aerosolizing procedure or had worn a mask or respirator under usual circumstances. High-risk HCWs are those who did not wear a mask during exposure or did not wear recommended PPE during an aerosolizing procedure [9].

Despite HCWs being at the epicenter of the pandemic, testing for HCWs and in-hospital digital tracing is not performed in most settings and there are no standardized tools present for HCWs. HCWs continue to work when they are asymptomatic due to the lack of testing and contact tracing; this leads to a vicious cycle of more patients and other HCWs being infected so it is extremely important to have effective contact tracing methods which could lead to not only early detection but also asymptomatic cases. Therefore, this study aims to describe the contact tracing experience that was initiated using an automated tool at King Faisal Specialist Hospital and Research Center (KFSH&RC), Saudi Arabia, where the tool was specifically dedicated just to HCWs and not the general population. 

## Materials and methods

An automated tool was developed to assist in the contact tracing process and was intended to be used by KFSH&RC employees who had been in contact with a COVID-19-positive individual for the early detection of possible COVID-19 cases. The tool categorized information into three separate groups containing the exposed employee and the index case who already had a positive COVID-19 test. The tool was made accessible to all of its 14,650 employees through the KFSH&RC official website and would require the employee's login details such as username and password, thereby preventing any unauthorized access or false entries. It collects information about 1) exposed employee information: ID badge number of the exposed individual, name, contact number, department, and medical record number and 2) index infected person information: ID/medical records number (MRN) of the index case, name of the index case, and department.

A risk categorization survey is a survey that comprises seven questions asked to categorize the contacts into three risk categories: no risk, low risk, and high risk. The risk is determined by the distance from the index case, area (closed or open), wearing a mask or not, time spent with the index case (20 minutes), and eating with an index case. Once this questionnaire form was submitted, the contact case would immediately receive an email indicating the risk category and what action to take based on the risk category.

The study includes data from June 2, 2020 (when the tool was implemented) to November 30, 2020, which was during the beginning of the pandemic.

**Figure 1:**
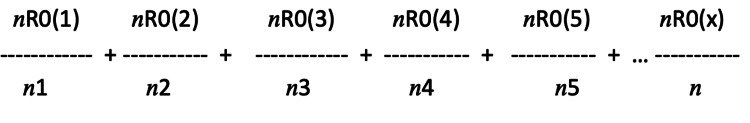
The basic reproduction number estimation equation 𝑛: The number of individuals in a specific group of the number of contacts R0: The average of positivity in a specific group of the number of contacts

The basic reproduction number (R0) is known as the average number of positive contact cases caused by an index case [10]. In this paper, the mode R0 is estimated by taking the average of positive cases based on the number of contacts for an index case, i.e., counting how many index cases had one contact, and then calculating the average of positivity among them similarly with index cases that had two contacts. The calculation of the mode is explained in Figure 1.

The data were coded and analyzed using the JMP software package, version 15 (SAS Institute Inc., Cary, NC). Chi-square test was used to compare the risk category and PCR result. Descriptive statistics for continuous variables were reported as mean and standard deviation. Categorical variables were summarized as frequencies and percentages. A p-value of 0.05 was considered statistically significant.

The identity of participants was kept confidential. Waiver of informed consent was found to be appropriate for this study. Ethical approval from the KFSH&RC Ethics Committee was obtained prior to data collection and analysis (approval No: 2221157).

## Results

**Figure 2:**
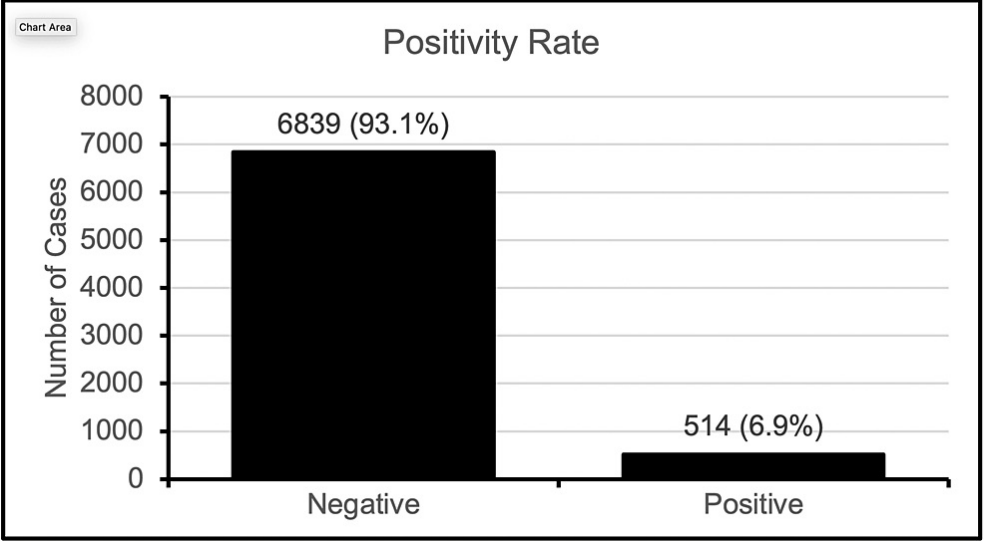
Polymerase chain reaction (PCR) results of contact cases

The tool has been utilized 7,353 times by contact cases since the development of this tool on June 2, 2020 until November 30, 2020. Figure 2 shows the positivity rate of contact tracing. Approximately 7% of those tested later became positive.

Table 1 shows the positivity frequency among the affected departments. The Environmental Services Department had the highest positivity rate per department (28.21%), followed by Health Information Technology and Analytics (HITA) (20%) and then Central Transportation (16.67%).

**Table 1:** Number of contacts and positive cases among departments

Level	Contact	Positive	Percentage of positive results
Physicians	1125	31	2.75%
Administration	531	31	5.83%
Case Management	4	0	0%
Central Transportation	56	9	16%
Clinical Services	1314	51	3.88%
Communication	13	0	0%
Critical Care Nursing	1	0	0%
Environmental Services	534	147	27.5%
Food Services	215	31	14.41%
HITA	10	2	20%
Housing Services	83	7	8.43%
Medical Stores	122	10	8.19%
Nursing	3037	90	2.96%
Project Management	91	6	6.59%
Safety & Security	202	17	8.14%
Supply Chain Management	82	9	10.97%
Transportation	24	1	4.16%
Utilities & Maintenance	521	72	13.81%
Total	7353	514	6.9%

There is a significant difference between the risk category and PCR results (p-value < 0.0001) (Table 2). The probability of having a positive result is greater associated with the high-risk group (13.8%).

**Table 2:** Comparing polymerase chain reaction (PCR) results with risk category

Risk Category	Result n (%)			P-value
	Negative	Positive	Total	
Low	4478 (97)	136 (3)	4614 (63)	<0.0001
High	2360 (86)	378 (14)	2738 (37)	
Total	6839	514	7353	

The average of the positives and the mode R0 obtained for the data using the equation described above was 0.30. The R0 per number of contacts who had index cases is described in Table 3.

**Table 3:** The average of positives and the mode R0 R0: basic reproduction number

Frequency of contact	Number of index cases	Average number of positives
1	628	0.13375796
2	205	0.2
3	157	0.1910828
4	120	0.35
5	78	0.56410256
6	54	0.33333333
7	50	0.8
8	36	0.91666667
9	26	0.34615385
10	29	0.82758621
11	17	0.17647059
12	16	0.25
13	14	0.21428571
14	13	1.07692308
15	11	0.27272727
16	5	0.6
17	10	0.4
18	8	0.875
19	6	1.33333333
20	3	0.33333333
21	6	0.16666667
22	4	1
23	4	1
24	5	1
25	3	3.33333333
26	3	1
27	4	0
28	3	0
29	3	2
30	2	2
31	2	0
32	3	0.66666667
33	4	0.75
35	1	4
37	1	2
40	1	2
41	1	2
43	1	0
49	1	0
53	1	3
58	1	0
85	1	0
89	1	2
92	1	0
208	1	0

## Discussion

In the COVID-19 pandemic, expedient identification of individuals with significant exposure to COVID-19 patients is a key strategy in breaking the chain of transmission and flattening the epidemiological curve [11]. Contact tracing is a systematic approach to identifying individuals who have been in contact with an infected person. It is a cornerstone of communicable disease containment and involves identifying, quarantining, and monitoring contacts of infected people [12]. Although in theory contact tracing is a known evidence-based strategy that has been extremely useful in containing several other infectious diseases in the community setting, the COVID-19 pandemic highlighted the challenges to implementing labor-intensive contact tracing, especially in the occupational setting of large healthcare systems and hospitals, the very epicenter of the pandemic [13]. Improving our understanding of the effectiveness of contact tracing in controlling diseases has substantial importance in public health and in preventing future pandemics.

While several governments around the world have implemented contact tracing and other versions of the program, there is still a scarcity when it comes to contact tracing in the healthcare sector. Similarly, there is a notable scarcity of documented data pertaining to HCWs in Saudi Arabia and their COVID-19 infection rates. To help control the spread of COVID-19 and ensure the safety of individuals, KFSH&RC, in collaboration with Infection Control and HITA, developed an automated staff COVID-19 contact tracing tool. This automated tool was not only developed to assist in the contact tracing process but also to make the process faster and not add further load on HCWs which would have been the case with manual contact tracing. A study conducted at a university hospital in Germany concluded that web-based contact tracing not only reduced the workforce required to conduct manual contact tracing but also offered fast test results as well as structured and comprehensive contact tracing for hospital employees [14]. From June 2, 2020 to November 30, 2020, the contact tracing tool at KFSH&RC had been utilized 7,965 times, of which 514 (6.9%) and 6,839 (93.1%) yielded a positive and negative PCR test, respectively. Among the 514 positive PCR results, 147 of them were from the environmental services department, constituting the highest number of positive PCRs, followed by the nursing department with 90 positive PCR cases. Lastly, the Utilities and Maintenance department experienced a total count of 72 positive PCR cases. This data infers that contrary to popular belief, most of the positive cases were not amongst primary healthcare providers and the medical team. This can mainly be attributed to the strict protocols involving personal protective equipment, which protect the primary medical team from any possible infections, thereby making them a low-risk category of exposure. This further emphasizes the importance of contact tracing to have a more targeted response by highlighting departments and areas that had the highest positivity. This can be achieved through stricter implementation of protocols as well as increasing awareness and education regarding the spread of the infection.

When looking at the data correlating the risk category and the likelihood of a positive PCR result, there is a strong statistical correlation between high-risk category exposures and positive PCR (p-value < 0.0001). Of the previously mentioned 514 positive cases, 378 (73.5%) of them were high-risk categories. Additionally, of the 6,838 negative cases, 4,478 (65.5%) of them were low-risk groups. The KFSH&RC COVID-19 tracing tool has highlighted its success by tracing and detecting 514 positive cases (in a timely manner that would have otherwise gone undetected) and a cumulative total of 7,965 index cases. According to the current hospital guidelines, low-risk exposure HCWs if asymptomatic can continue working if they adhere to strict safety protocols. In addition, for high-risk exposures, HCWs are required to strictly adhere to N95 masks in the health care facility, even when lounging during their break time, and are required to test on the third to the fifth day of exposure and refrain from working if the test result is positive [9]. HCWs who report their symptoms to their institution are mandated to get tested and quarantined. Instructions for testing their family members or housemates are similar to the general population, while other co-workers are asked to monitor for symptoms [9]. The implementation of the hospital’s contact-tracing process also supported comprehensive down streaming of contact-tracing activities as well as improved manpower utilization both in contact tracing as well as the workforce by early detection, risk categorization, and early implementation of safety protocols.

Some of the limitations of the study include the limited variability of patients. The individuals traced were all part of the hospital system. Therefore, the observed outcomes and results have low generalizability. Second, the positive results among contacts might have been biased because the data was not regularly collected for all contacts. Testing of contacts, specifically asymptomatic contacts, might not have been available and could have been further confounded by the recommendations relating to the importance of testing all contacts.

## Conclusions

This study acts as the first of its kind to describe the successful use of the healthcare contact tracing system in one of Saudi Arabia's largest hospitals - KFSH&RC. Using this tool, the hospital was not only able to handle the increased number of index cases during the rapidly evolving pandemic between June 2020 and November 2020 but also describe the infection trends in its different departments. Departments with the highest number of positive COVID-19 cases included Environmental Services, HITA, and Central Transportation, in that order. Contrary to popular belief, physicians and nursing staff had one of the lowest positivity rates. This can be attributed to the hospital’s strict PPE policy and the medical staff’s adherence to the same. After its launch, the tracing system has managed to prove its reliability by screening a total of 7,353 cases, from which 557 (6.9%) of the cases traced later on developed a COVID-19 infection. However, to manage rapidly changing situations like future pandemics, further development of technical solutions for contact tracing in healthcare settings is required.
